# Supplementary material for: Metformin alters mitochondria-related metabolism and enhances human oligodendrocyte function
Source: Nat Commun. 2025 Aug 30;16:8126. doi: 10.1038/s41467-025-63279-4 (PMC12398550; doi:10.1038/s41467-025-63279-4)
Supplement: Supplementary file 1 — Supplementary Information [file 41467_2025_63279_MOESM1_ESM.pdf]

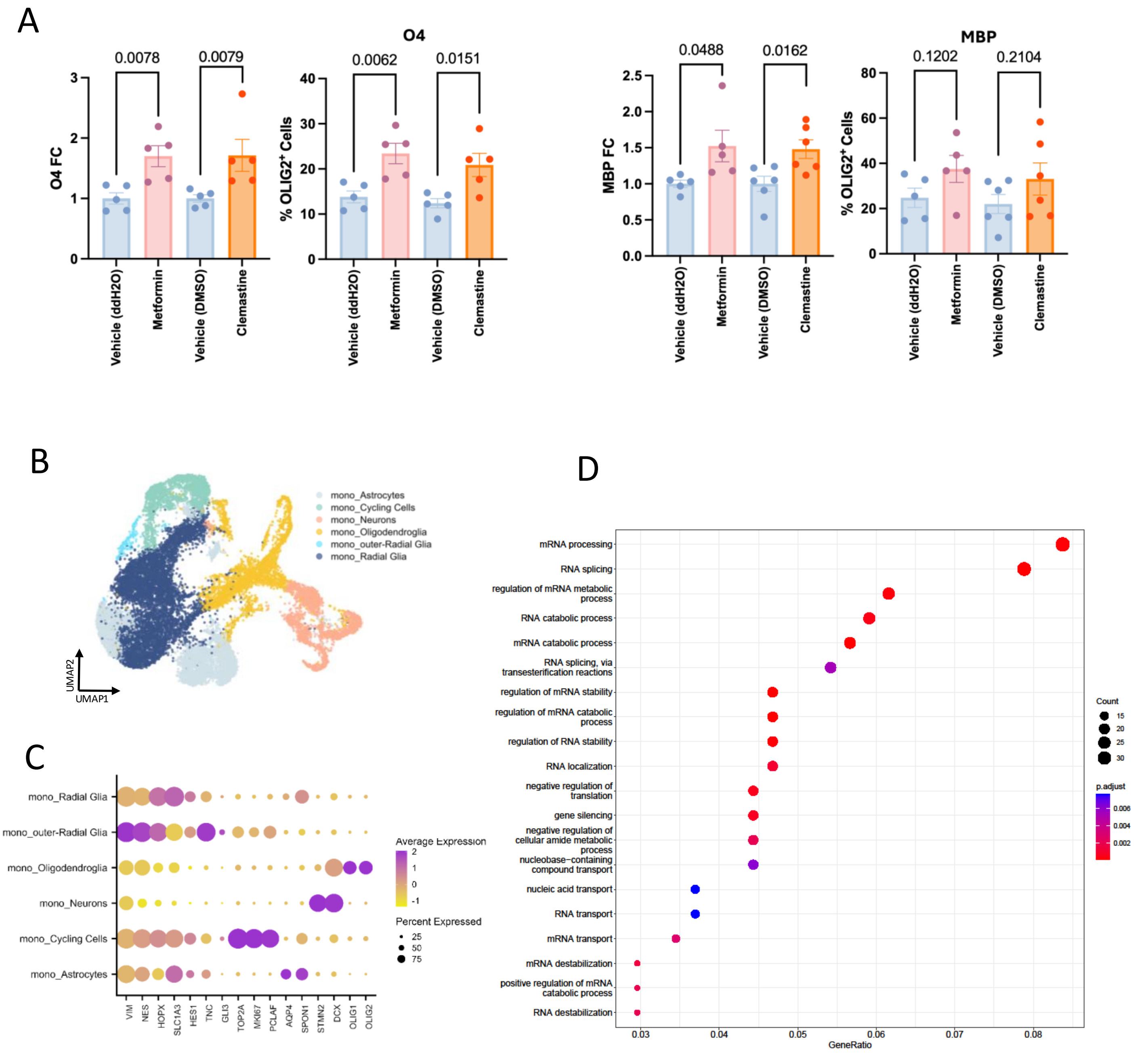

Supplementary Figure 1: hESC-derived monolayer oligodendroglia cultures

A) Fold change (FC) difference or percentages of OLIG2$^+$O4$^+$ or OLIG2$^+$MBP$^+$ oligodendrocytes after treatment with metformin or clemastine compared to their respective vehicle-treated controls (ddH$_2$O or DMSO). n = 5 (O4) or n=6 (MBP) differentiations with 4 technical repeats for each. Kolmogorov-Smirnov normality test with Dallal-Wilkinson-Lillie for p value, two-tailed unpaired t-test. Mean ± SEM. (B) UMAP representation of the complete dataset showing broad cell types. (C) Dot plot of selected marker genes showing different cell lineages. (D) GO analysis of differentially expressed genes of the metabolically active oligodendroglia (MAO) sub-cluster. Analysis was performed on differentially expressed genes (p_adj ≤ 0.05, fold-change expressed logarithmically (logFC) ≥ 0.5).

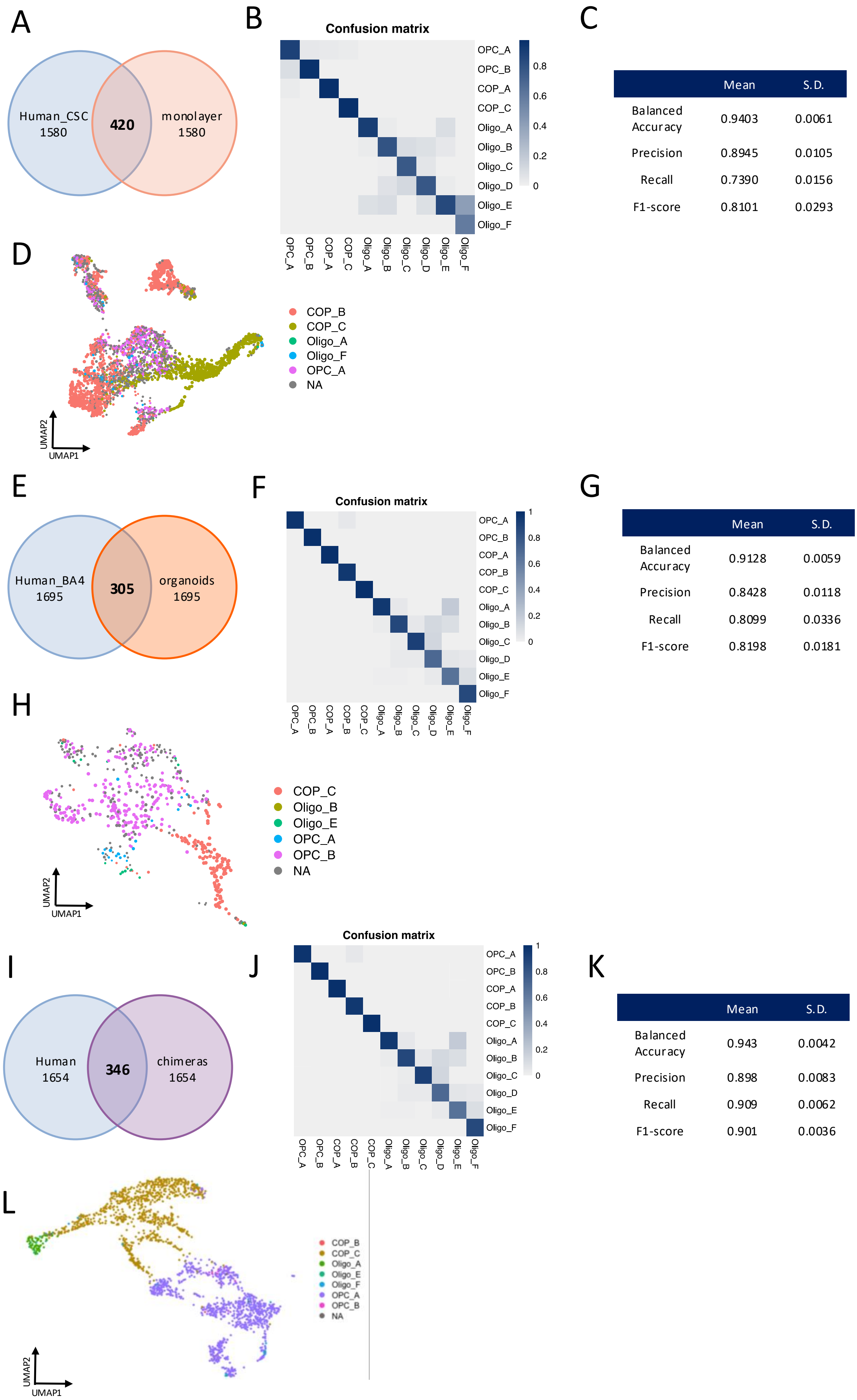

	Mean	S.D.
Balanced Accuracy	0.9403	0.0061
Precision	0.8945	0.0105
Recall	0.7390	0.0156
F1-score	0.8101	0.0293

	Mean	S.D.
Balanced Accuracy	0.9128	0.0059
Precision	0.8428	0.0118
Recall	0.8099	0.0336
F1-score	0.8198	0.0181

	Mean	S.D.
Balanced Accuracy	0.943	0.0042
Precision	0.898	0.0083
Recall	0.909	0.0062
F1-score	0.901	0.0036

Supplementary Figure 2: Performance of ANN classifier and label transfer result. (A) The ANN was trained to recognize single-cell gene expression profiles of human spinal cord oligodendroglia using the union of variable highly variable features between human post mortem oligodendroglia and hESC-derived monolayer oligodendroglia. (B) Confusion matrix of the true and predicted labels in the source domain (human data) for monolayer dataset. (C) Performance metrics in the source domain (balanced accuracy, precision, recall) for monolayer dataset. (D) UMAP visualization of scRNAseq data with label transferred from the human dataset to the monolayer dataset using ANN. (E) The ANN was trained to recognize single-cell gene expression profiles of human cortical oligodendroglia using the union of variable highly variable features between human and hESC-derived organoid oligodendroglia. (F) Confusion matrix of the true and predicted labels in the source domain (human data) for organoid dataset. (G) Performance metrics in the source domain (balanced accuracy, precision, recall) for monolayer dataset. (H) UMAP visualization of scRNAseq data with label transferred from the human dataset to the organoid dataset using ANN. (I) The ANN was trained to recognize single-cell gene expression profiles of human oligodendroglia using the union of highly variable features between human and hESC-derived oligodendroglia from chimeras. (J) Confusion matrix of the true and predicted labels in the source domain (human data) for chimeric dataset. (K) Performance metrics in the source domain (balanced accuracy, precision, recall) for monolayer dataset. (L) UMAP visualization of scRNAseq data with labels transferred from the human dataset to the chimeric dataset using ANN.

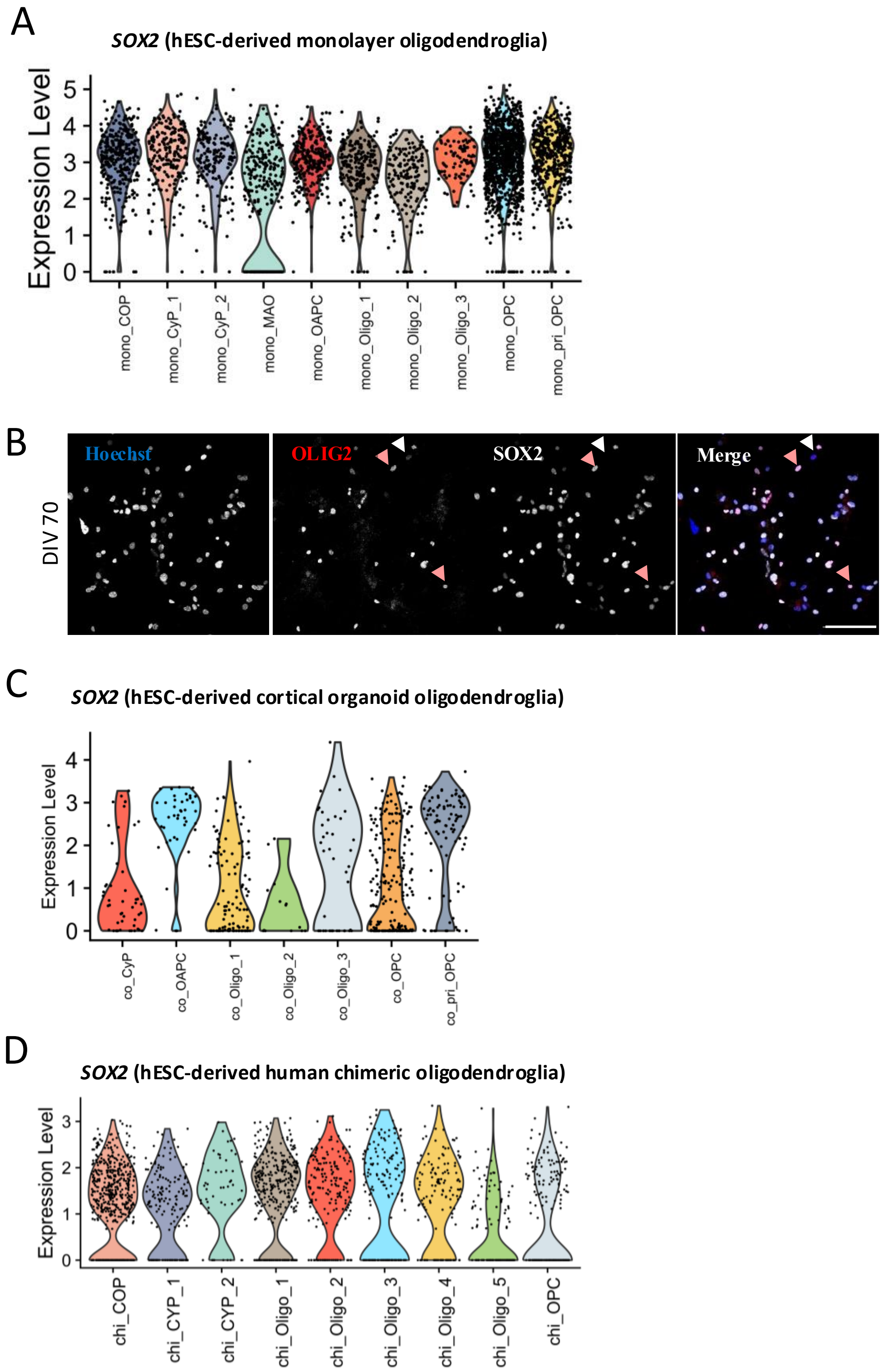

Supplementary Figure 3: *SOX2* expression persists in hESC-derived oligodendroglia whether cultured in monolayer, organoids or in chimeras

Violin plot showing expression of *SOX2* in (A) hESC-derived monolayer oligodendroglia. (B) Immunofluorescence image of hESC-derived oligodendroglia at day 70 of in vitro differentiation (DIV) showing OLIG2 (red) and SOX2 (grey) co-expression at the protein level. Red arrowheads indicate $OLIG2^{+}SOX2^{+}$ cells, while white arrowheads indicate $OLIG2^{+}SOX2^{-}$ cells. Scale bar = 100µm. Violin plots showing expression of *SOX2* in (C) hESC-derived cortical brain organoid oligodendroglia and (D) hESC-derived chimeric oligodendroglia.

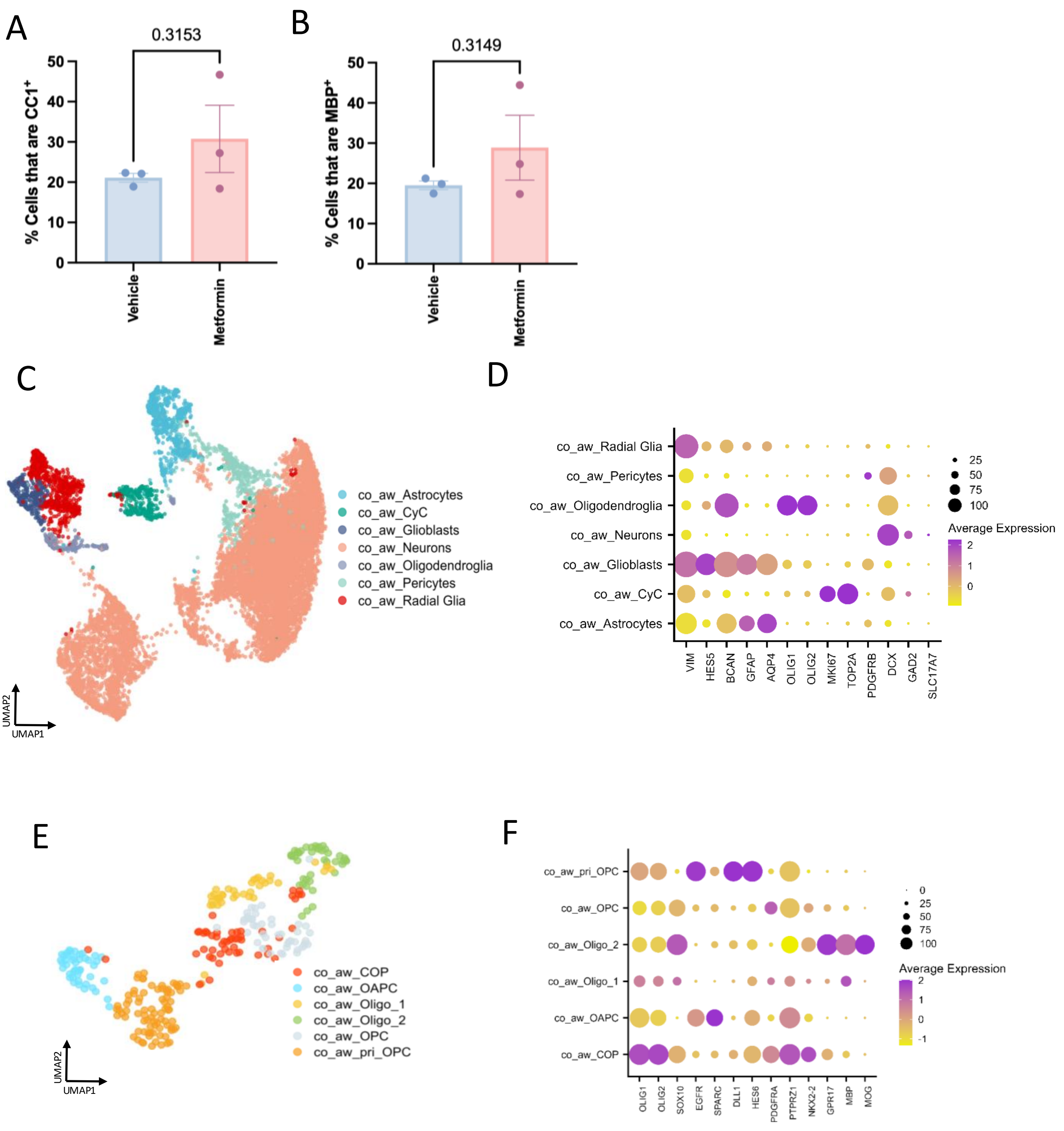

Supplementary Figure 4: hESC-derived cultured cortical organoid oligodendroglia

A) Percentage of cells in spheroids that are mature $CC1^+$ oligodendrocytes with and without metformin treatment. Two-tailed unpaired t-test, mean ± SEM. B) Percentage of cells in spheroids that are mature MBP^+ oligodendrocytes with and without metformin treatment. N=3 cultures. Two-tailed unpaired t-test, mean ± SEM. C) UMAP representation of the complete organoid dataset showing broad cell types. (D) Dot plot of selected marker genes showing different cell lineages. (E) UMAP representation of clustered oligodendroglia. (F) Dot plot of selected marker genes showing cluster segregation.

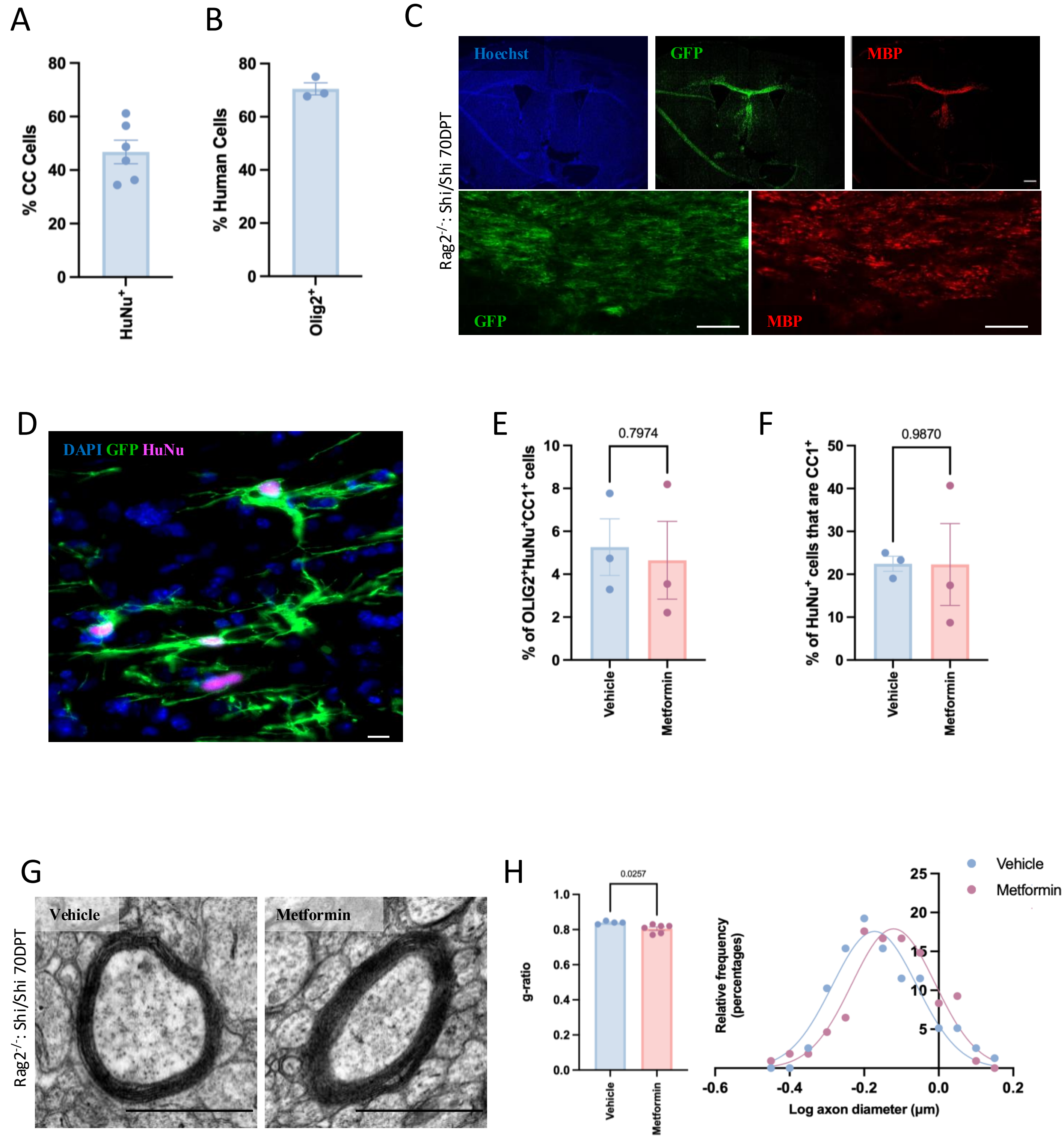

Supplementary Figure 5: Characterisation of hESC-derived chimeric tissue oligodendroglia after metformin treatment.

(A) Percentage of hESC-derived HuNu$^+$ cells (n=6 mice), and (B) HuNu$^+$OLIG2$^+$ cells (oligodendroglial lineage)(n=3 mice) in the chimeric mouse corpus callosum 70 days post transplantation. Mean ± SEM. (C) Immunofluorescence showing MBP (myelin and oligodendrocytes, red), GFP (membrane tagged so in myelin, green) and Hoechst (nuclei, blue) of coronal sections of mouse brain with corpus callosum transplanted with GFP$^+$ hESC-derived oligodendroglia. Upper, overview, scale bar = 2mm, lower, zoom into corpus callosum, scale bar = 500um. (D) High resolution image of GFP$^+$ transplanted human (HuNu$^+$, magenta) oligodendrocytes in mouse corpus callosum (DAPI=nuclei, blue). Scale bar = 10um. (E) Percentage of all cells in corpus callosum region which are OLIG2$^+$HuNu$^+$CC1$^+$ oligodendrocytes, and (F) of all human oligodendroglia which are CC1$^+$ in the chimeric mouse corpus callosum 70 days post transplantation comparing control and metformin-treated groups. Points are individual mice (n=3 mice), two-tailed unpaired t-test, mean ± SEM. (G) EM of single axons in the corpus callosum of *Shi/Shi:Rag2*$^{-/-}$ chimeric mice, 21 days post metformin or vehicle treatment, demonstrating thickness of compact human myelin. Scale bar = 1µm. (H) Treatment with metformin decreases average g-ratio (thicker myelin) compared to vehicle-treated chimeras, while axonal diameter (log-transformed values) remains unchanged. n = 6 metformin animals, n = 4 vehicle-treated animals with ~15-20 axons analysed per animal. Kolmogorov-Smirnov normality test with Dallal-Wilkinson-Lillie for p value, two-tailed unpaired t-test. Mean ± SEM.

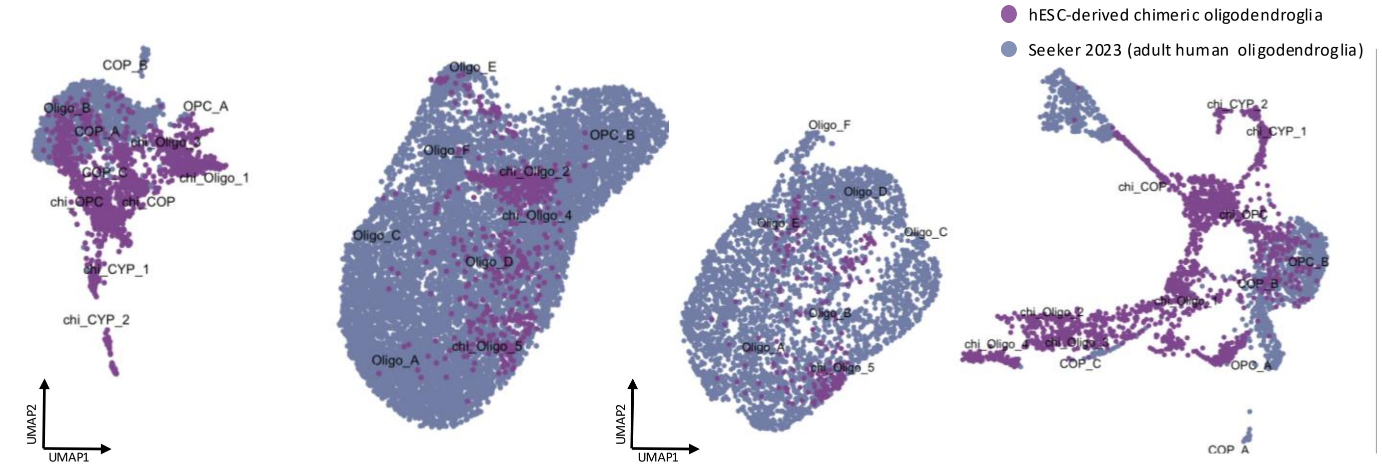

CCA of oligodendroglia from HCA Spinal cord dataset and chimeras

CCA of oligodendroglia from HCA brain white matter dataset and chimeras

Supplementary Figure 6: UMAP plots illustrating CCA of oligodendroglia from the HCA Spinal cord snRNAseq dataset or the brain white matter dataset (Seeker et al., 2023) with human oligodendroglia extracted and undergoing scRNASeq from our chimeric mice model. Purple – chimera oligodendroglia, grey – HCA snRNAseq data.

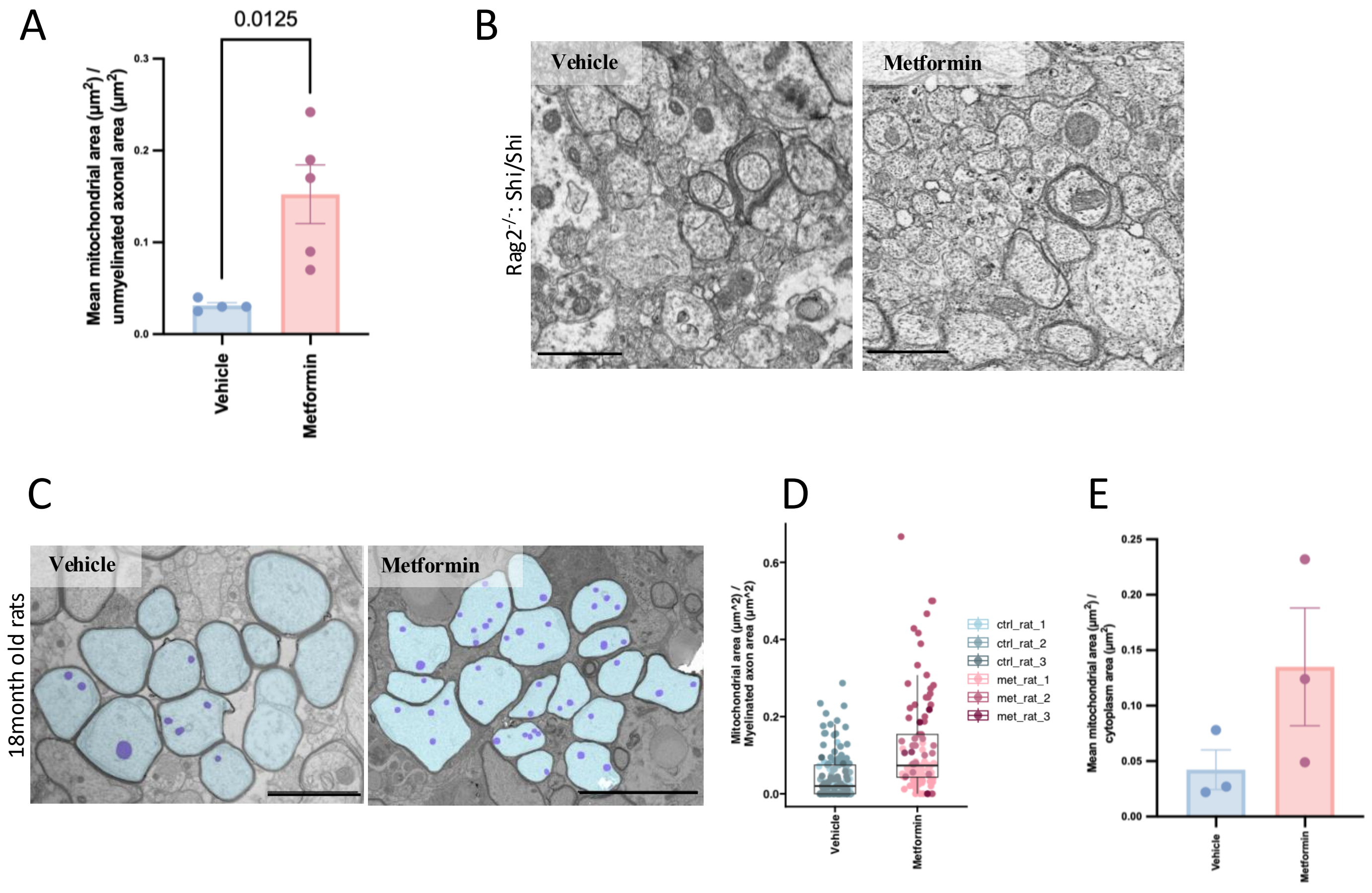

Supplementary Figure 7: (A) Significant increase of mitochondrial area in axons without compact myelin (similar to those with myelin) after metformin treatment (n=5 mice) compared to vehicle-treated controls (n=4 mice) (average per mouse), means ± SEM. (B) *Shi/Shi:Rag2*$^{-/-}$ mice without human ES-derived oligodendroglia transplantation do not show compact myelin profiles, either treated with water or metformin. Scale bar = 1µm. (C) EM of aged (18mth old) rat brains show (D,E) a similar trend to increased mitochondrial area within axons after treatment with metformin. Points are individual axon values (D), average per rat (E), n = 3 metformin- and n = 3 vehicle-treated animals. Two-tailed unpaired, mean ± SEM. Boxes in plots visualize median and 25th and 75th percentiles and whiskers mark range up to 1.5 * inter-quartile ranges to show potential outliers. See Supplemental Table 1 for exact summary statistics.

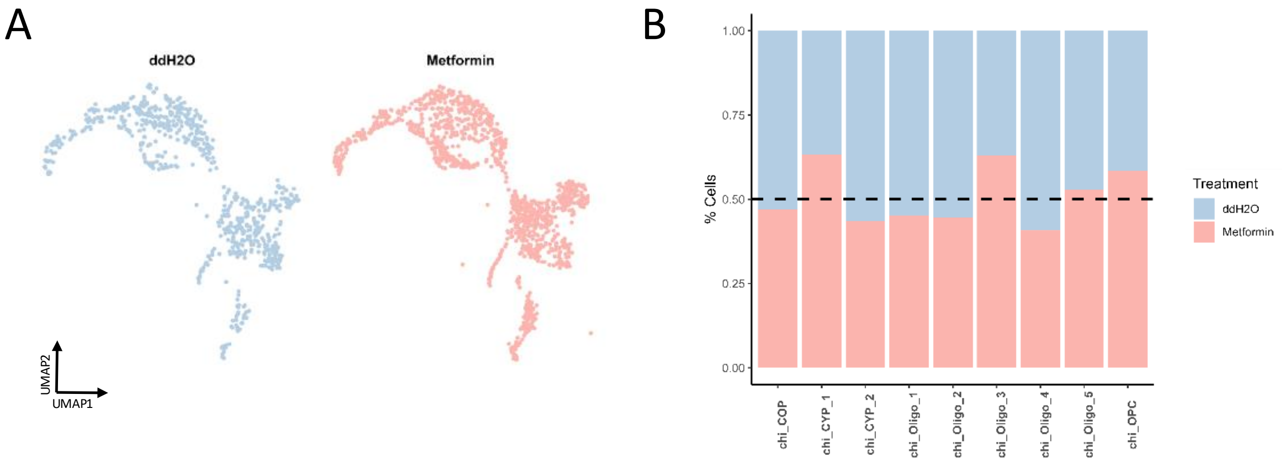

Supplementary Figure 8: hESC-derived oligodendroglia from chimeric mice do not demonstrate compositional alterations after metformin treatment. (A) UMAP representation of the complete dataset shows no selective clusters and (B) no marked compositional changes after treatment metformin.

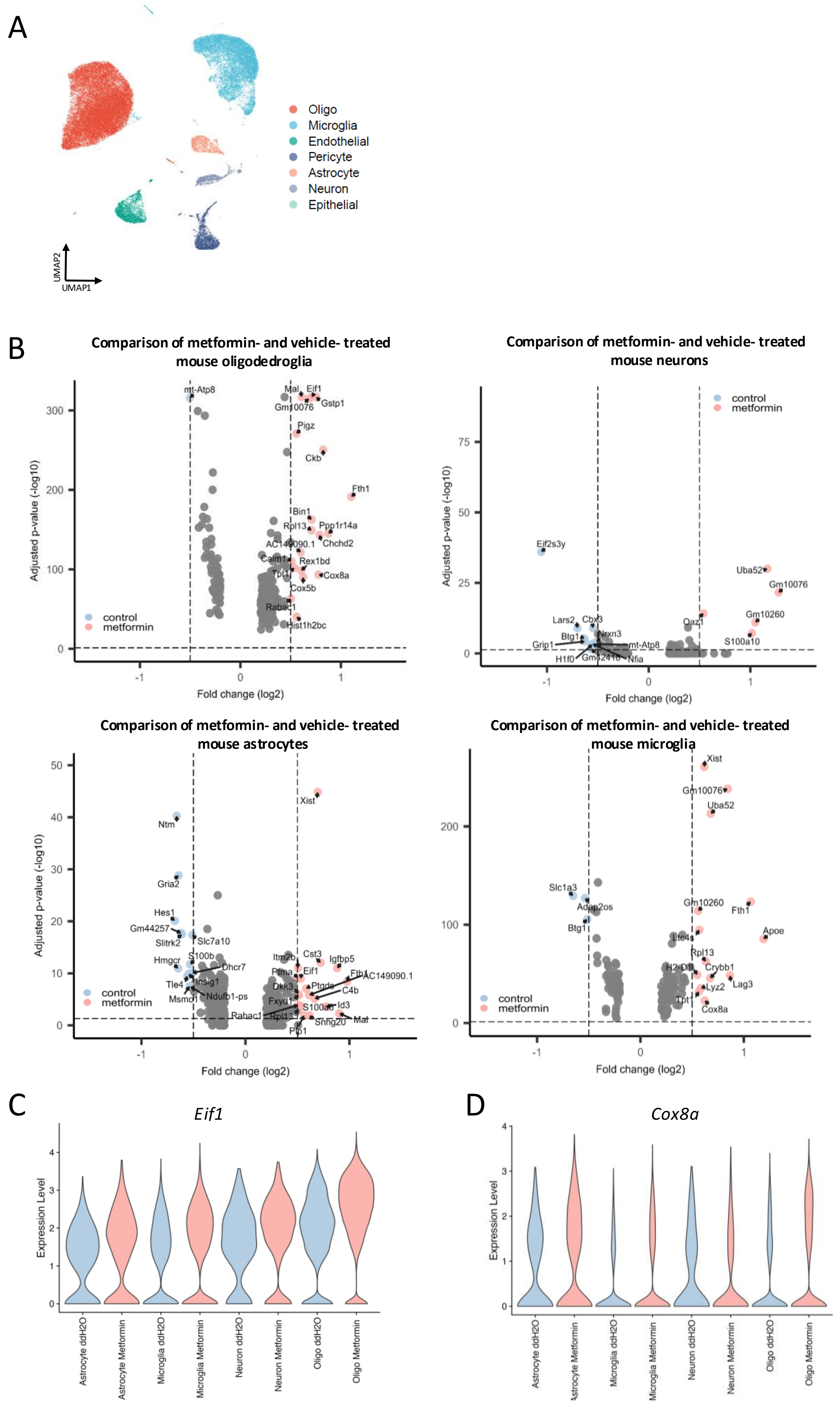

Supplementary Figure 9: Mouse cells from chimeras demonstrate similar changes to human cells after metformin treatment. (A) UMAP representation of mouse cells from the corpus collosum showing different cell lineages. (B) Volcano plots of significantly differentially expressed genes between mouse oligodendroglia, neurons, astrocytes and microglia from metformin- and vehicle-treated chimeras. (C) Violin plot showing expression of *Eif1* and (D) *Cox8a* metformin- and vehicle-treated mouse oligodendroglia, astrocytes, microglia and neurons.

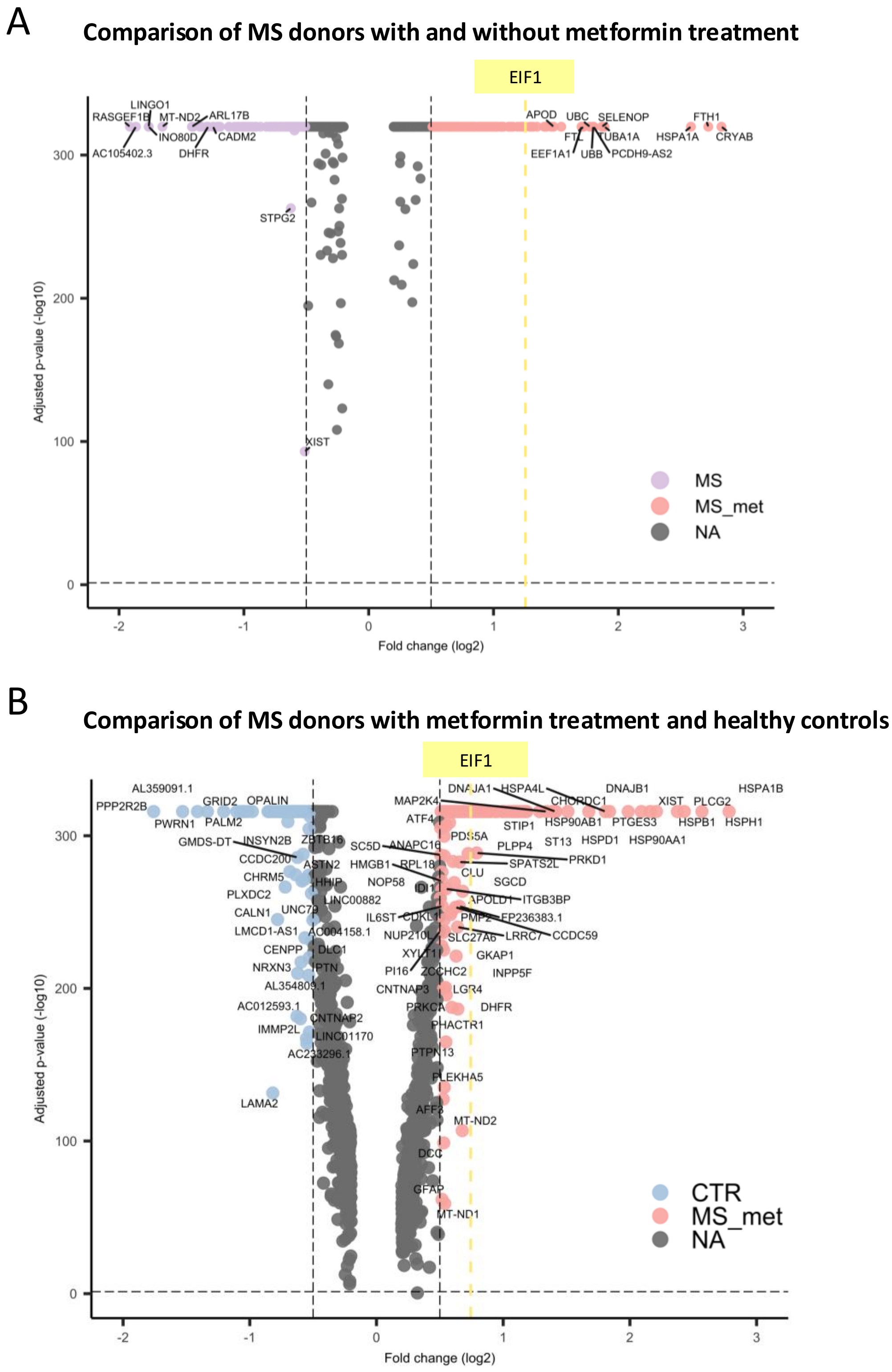

Supplementary Figure 10: *EIF1* is upregulated in oligodendrocytes from human MS donors exposed to metformin. (A) Volcano plot showing upregulated genes and specific upregulation of *EIF1* in MS donors on metformin compared to MS donors not on metformin and (B) showing upregulated genes and specific upregulation of *EIF1* in MS donors on metformin treatment compared to healthy controls (dashed yellow line and highlighted).
