# Supplementary material for: Metformin alters mitochondria-related metabolism and enhances human oligodendrocyte function
Source: Nat Commun. 2025 Aug 30;16:8126. doi: 10.1038/s41467-025-63279-4 (PMC12398550; doi:10.1038/s41467-025-63279-4)
Supplement: Supplementary file 2 — Description of Additional Supplementary Files [file 41467_2025_63279_MOESM2_ESM.pdf]

Description of Additional Supplementary Files

Supplementary Data S1 - DE analysis of metformin- and vehicle-treated groups (chimeric human cells)

Supplementary Data S2 - DE analysis of metformin- and vehicle-treated groups (chimeric mouse oligodendroglia)

Supplementary Data S3 - DE analysis of metformin- and vehicle-treated groups (chimeric mouse astrocytes)

Supplementary Data S4 - DE analysis of metformin- and vehicle-treated groups (chimeric mouse microglia)

Supplementary Data S5 - DE analysis of metformin- and vehicle-treated groups (chimeric mouse neurons)

Supplementary Data S6 - DE analysis of metformin-treated MS patients and healthy controls

Supplementary Data S7 - DE analysis of metformin-treated and untreated MS patients

Supplementary Data S8 - Reagents for culturing hESCs, monolayer oligodendrocytes and cortical organoids

Supplementary Data S9 - Primary antibodies

Supplementary Data S10 - CNV analysis

Supplementary Data S11 - Box and whisker plot values for Figure 4B, 4D and S7D
