# Supplementary material for: Metformin alters mitochondria-related metabolism and enhances human oligodendrocyte function
Source: Nat Commun. 2025 Aug 30;16:8126. doi: 10.1038/s41467-025-63279-4 (PMC12398550; doi:10.1038/s41467-025-63279-4)
Supplement: Supplementary file 4 — Reporting Summary [file 41467_2025_63279_MOESM4_ESM.pdf]

nature portfolio

Corresponding author(s): Anna Williams

Last updated by author(s): Jul 24, 2025

Reporting Summary

Nature Portfolio wishes to improve the reproducibility of the work that we publish. This form provides structure for consistency and transparency in reporting. For further information on Nature Portfolio policies, see our Editorial Policies and the Editorial Policy Checklist.

Statistics

For all statistical analyses, confirm that the following items are present in the figure legend, table legend, main text, or Methods section.

n/a	Confirmed	
☐	☒	The exact sample size (*n*) for each experimental group/condition, given as a discrete number and unit of measurement
☐	☒	A statement on whether measurements were taken from distinct samples or whether the same sample was measured repeatedly
☐	☒	The statistical test(s) used AND whether they are one- or two-sided<br>*Only common tests should be described solely by name; describe more complex techniques in the Methods section.*
☐	☒	A description of all covariates tested
☐	☒	A description of any assumptions or corrections, such as tests of normality and adjustment for multiple comparisons
☐	☒	A full description of the statistical parameters including central tendency (e.g. means) or other basic estimates (e.g. regression coefficient) AND variation (e.g. standard deviation) or associated estimates of uncertainty (e.g. confidence intervals)
☐	☒	For null hypothesis testing, the test statistic (e.g. *F*, *t*, *r*) with confidence intervals, effect sizes, degrees of freedom and *P* value noted<br>*Give P values as exact values whenever suitable.*
☒	☐	For Bayesian analysis, information on the choice of priors and Markov chain Monte Carlo settings
☒	☐	For hierarchical and complex designs, identification of the appropriate level for tests and full reporting of outcomes
☒	☐	Estimates of effect sizes (e.g. Cohen's *d*, Pearson's *r*), indicating how they were calculated

Our web collection on statistics for biologists contains articles on many of the points above.

Software and code

Policy information about availability of computer code

Data collection	Immunofluorescence was imaged on a wide field Zeiss observer using Zeiss Zen (blue edition), a Leica TCS SP8 confocal or the Opera Phenix High-Content Screening System. TEM images were acquired on a Jeol JEM1400 plus Transmission Electron Microscope with OneView Camera. Single cell RNA sequencing libraries were sequenced using a NovaSeq 6000 sequencingsystem (PE150 (HiSeq), Illumina). Western blot protein bands were visualised and imaged with a ChemiDoc MP Imaging System (Bio-Rad).
Data analysis	Manual cell counts were performed using Fiji/ImageJ (v1.54). Semi-automated counts were performed using Qupath software (v0.3.0). MBP+ area quantification was automatically analysed using a Fiji/ImageJ macro. g-ratios were measured using the TrackEM plugin in Fiji/ImageJ. Data handling was managed on Microsoft Excel. Statistical analysis was performed using Graphpad Prism (8.3.0). Single-cell RNA sequences were aligned to the reference genome, feature counting and cell calling were performed following the STARSolo (STAR version 2.7.8a) or 10x Genomics CellRanger (v.5.0.0) pipeline with the human and mouse reference genomes provided by 10x: GRCh38 2020-A, mm10 2020-A . The downstream analysis was performed in R, with QC performed with scater using the isOutlier function for library size and mitochondrial gene filtering. Genes expressed in ≤ 10 cells were also removed. Data normalization and feature variance estimation were performed using Scran, using logNormCounts() and modelGeneVar(), while dimensional reduction and clustering were performed with Seurat' v4 implementation of the Louvain algorithm. The clusters were annotated using canonical markers and lists of differentially expressed markers between clusters. Differential gene expression analysis for the identification of cluster marker genes and genes altered after treatment with metformin were performed with MAST using Seurat's FindMarkers() wrapper and filtering genes that had a minimum positive log2-fold change (log2FC) of 0.25 and were expressed by at least 20% of cells within the cluster/group of interest and less than 60% outwith. When comparing metformin-treated and control conditions, additional thresholds of absolute log2FC of ≥ 0.5 and adjusted p-value of < 0.05 were applied.

For manuscripts utilizing custom algorithms or software that are central to the research but not yet described in published literature, software must be made available to editors and reviewers. We strongly encourage code deposition in a community repository (e.g. GitHub). See the Nature Portfolio guidelines for submitting code & software for further information.

Data

Policy information about availability of data

All manuscripts must include a data availability statement. This statement should provide the following information, where applicable:

- Accession codes, unique identifiers, or web links for publicly available datasets
- A description of any restrictions on data availability
- For clinical datasets or third party data, please ensure that the statement adheres to our policy

The data that support the findings in this study are available as unrestricted source data files. Our RNAseq data is open access already and available at GEO with Accession number: GSE282369.

Research involving human participants, their data, or biological material

Policy information about studies with human participants or human data. See also policy information about sex, gender (identity/presentation), and sexual orientation and race, ethnicity and racism.

Reporting on sex and gender	N/A
Reporting on race, ethnicity, or other socially relevant groupings	N/A
Population characteristics	N/A
Recruitment	N/A
Ethics oversight	N/A

Note that full information on the approval of the study protocol must also be provided in the manuscript.

Field-specific reporting

Please select the one below that is the best fit for your research. If you are not sure, read the appropriate sections before making your selection.

[x] Life sciences [ ] Behavioural & social sciences [ ] Ecological, evolutionary & environmental sciences

For a reference copy of the document with all sections, see nature.com/documents/nr-reporting-summary-flat.pdf

Life sciences study design

All studies must disclose on these points even when the disclosure is negative.

Sample size	This was a hypothesis driven study. Sample sizes were based on ours and other's previous studies (Livesey et al., 2016, Marton et al., 2019, Neumann et al., 2019, Boyd et al., 2013), estimating similar effect sizes.
Data exclusions	No data was excluded from the anaylsis
Replication	Technical and biological repeats are described in the text/methods/legends.
Randomization	Mice and cell samples were randomly assigned to each experimental group
Blinding	Experimentor was blinded during all data collection and analysis

Reporting for specific materials, systems and methods

We require information from authors about some types of materials, experimental systems and methods used in many studies. Here, indicate whether each material, system or method listed is relevant to your study. If you are not sure if a list item applies to your research, read the appropriate section before selecting a response.

Materials & experimental systems

n/a	Involved in the study	
☐	☒	Antibodies
☐	☒	Eukaryotic cell lines
☒	☐	Palaeontology and archaeology
☐	☒	Animals and other organisms
☒	☐	Clinical data
☒	☐	Dual use research of concern
☒	☐	Plants

Methods

n/a	Involved in the study	
☒	☐	ChIP-seq
☒	☐	Flow cytometry
☒	☐	MRI-based neuroimaging

Antibodies

Antibodies used

Rat monoclonal anti-MBP Serotec MCA409S RRID:AB_325004 (1:250)
Rabbit polyclonal anti-Olig2 Millipore AB9610 RRID:AB_570666 (1:400)
Goat polyclonal anti-Olig2 R&DSystems AF2418 RRID:AB_2157554 (1:400)
Mouse monoclonal anti-NucleiAntibody, clone235-1 Millipore MAB1281 RRID:AB_94090 (1:400)
Mouse monoclonal anti-Oligodendrocyte Marker O4 R&DSystems MAB1326 RRID:AB_357617 (1:400)
Rabbit monoclonal anti-PDGFRa Cellsignalling 3174 RRID:AB_2162345 (1:200)
Chicken monoclonal anti-MBP Encor CPCA-MBP (1:2000)
Mouse monoclonal anti-APC/CC1 Abcam ab16794 (1:400)
Rabbit polyclonal anti OLIG2 Atlas antibodies HPA003254 (1:200)
Rabbit polyclonal anti-TOMM20 Proteintech 11802-1-AP (1:2000)
Rabbit polyclonal anti-CHCHD2 Proteintech 19424-1-AP (1:1000)
Mouse monoclonal anti-B-TUBULIN Sigma T5293 (1:100)

Validation

Validation of the antibodies used were stated on the suppliers websites. Olig2 (HPA003254, Atlas Antibodies), MBP (CPCA-MBP, Encor), APC/CC1 (ab16792, Abcam), TOMM20 (11802-1-AP. Proteintech), CHCHD2 (19424-1-AP, Proteintech) and B-tubulin (clone 2-28-33, Sigma) showed signal in accordance with, respectively; Jakel et al. Nature (2019), Procacci et al. Glia (2023), Lee et al. PLoS One (2014), Cruz-Zaragoza et al. Cell (2021), and Feyeux & Bourgois-Rocha et al. Human Molecular Genetics (2012). Ortiz-Rivero et al Cell Commun Signal (2018).

Eukaryotic cell lines

Policy information about cell lines and Sex and Gender in Research

Cell line source(s)

RC17 GMP grade, female, human embroynic cell line was used - De Sousa et al. Stem Cell Research, (2016). Used with Human Stem cell authority permission.

Authentication

N/A

Mycoplasma contamination

absent

Commonly misidentified lines
(See ICLAC register)

N/A

Animals and other research organisms

Policy information about studies involving animals; ARRIVE guidelines recommended for reporting animal research, and Sex and Gender in Research

Laboratory animals

Shi/Shi:Rag 2-/- P2-4, and 10 weeks. Homozygous Shiverer mice (The Jackson Laboratory, Bar Harbor, ME, C3HeB background) were crossed with homozygous Rag2-null immunodeficient mice (The Jackson Laboratory, Bar Harbor, ME, C57/BL6J background) to generate Shi/Shi:Rag2-/- myelin-deficient, immunodeficient mice.

Wild animals

N/A

Reporting on sex

Mice of both sexes were used.

Field-collected samples

N/A

Ethics oversight

Experiments were performed under a UK Home Office Licence granted to Anna Williams with ethical approval.

Note that full information on the approval of the study protocol must also be provided in the manuscript.

Plants

Seed stocks	*Report on the source of all seed stocks or other plant material used. If applicable, state the seed stock centre and catalogue number. If plant specimens were collected from the field, describe the collection location, date and sampling procedures.*
Novel plant genotypes	*Describe the methods by which all novel plant genotypes were produced. This includes those generated by transgenic approaches, gene editing, chemical/radiation-based mutagenesis and hybridization. For transgenic lines, describe the transformation method, the number of independent lines analyzed and the generation upon which experiments were performed. For gene-edited lines, describe the editor used, the endogenous sequence targeted for editing, the targeting guide RNA sequence (if applicable) and how the editor was applied.*
Authentication	*Describe any authentication procedures for each seed stock used or novel genotype generated. Describe any experiments used to assess the effect of a mutation and, where applicable, how potential secondary effects (e.g. second site T-DNA insertions, mosiacism, off-target gene editing) were examined.*
